# A perspective study of the possible impact of obeticholic acid against SARS-CoV-2 infection

**DOI:** 10.1007/s10787-022-01111-x

**Published:** 2022-12-09

**Authors:** Gaber El-Saber Batiha, Hayder M. Al-kuraishy, Ali I. Al-Gareeb, Fadia S. Youssef, Suzy A. El-Sherbeni, Walaa A. Negm

**Affiliations:** 1 Department of Pharmacology and Therapeutics, Faculty of Veterinary Medicine, Damanhour University, Damanhour, 22511 AlBeheira Egypt; 2 Department of Clinical Pharmacology and Medicine, College of Medicine, ALmustansiriyia University, Baghdad, Iraq; 3 Department of Pharmacognosy, Faculty of Pharmacy, Ain-Shams University, Abbasia, Cairo, 11566 Egypt; 4 Department of Pharmacognosy, Faculty of Pharmacy, Tanta University, Tanta, 31527 Egypt

**Keywords:** Covid-19, Farnesoid X receptor, SARS-Cov-2 infection, Obeticholic acid

## Abstract

The causative agent of CoV disease 2019 is a new coronavirus CoV type 2, affecting the respiratory tract with severe manifestations (SARS-CoV-2). Covid-19 is mainly symptomless, with slight indications in about 85% of the affected cases. Many efforts were done to face this pandemic by testing different drugs and agents to make treatment protocols in different countries. However, the use of these proposed drugs is associated with the development of adverse events. Remarkably, the successive development of SARS-CoV-2 variants which could affect persons even they were vaccinated, prerequisite wide search to find efficient and safe agents to face SARS-CoV-2 infection. Obeticholic acid (OCA), which has anti-inflammatory effects, may efficiently treat Covid-19. Thus, the goal of this perspective study is to focus on the possible medicinal effectiveness in managing Covid-19. OCA is a powerful farnesoid X receptor (FXR) agonist possessing marked antiviral and anti-inflammatory effects. FXR is dysregulated in Covid-19 resulting in hyper-inflammation with concurrent occurrence of hypercytokinemia. Interestingly, OCA inhibits the reaction between this virus and angiotensin-converting enzyme type 2 (ACE2) receptors. FXR agonists control the expression of ACE2 and the inflammatory signaling pathways in this respiratory syndrome, which weakens the effects of Covid-19 disease and accompanied complications. Taken together, FXR agonists like OCA may reveal both direct and indirect impacts in the modulation of immune reaction in SARS-CoV-2 conditions. It is highly recommended to perform many investigations regarding different phases of the discovery of new drugs.

## Introduction

Wuhan, China, in December 2019 was the first place for detection of coronavirus disease with serious respiratory manifestations (Al-Kuraishy et al. 2021d). The World Health Organization (WHO) at the beginning of 2020 named it CoV disease 2019 (Al-Kuraishy et al. 2021c). SARS-CoV-2 utilizes special receptors to enter human cells and ACE2 is one of the main receptors (Onohuean et al. 2021). The binding of this pandemic virus to ACE2 results in a series of inflammatory cellular incidents with pathological consequences resulting in cell deterioration and augmented inflammation response. A few different cellular systems such as neurons, pulmonary alveolar cells, and cardiomyocytes have ACE2 in which it is widely expressed and distributed (Al-Kuraishy et al. 2020a; Elekhnawy and Negm 2022). It is notable that Covid-19 first introduced clinically asymptomatically, with reasonable symptoms occurring in approximately 85% of affected people. Manifestation of symptoms may be moderate-to-severe due to the production of acute lung damage recognized in 15% of cases (ALI). Furthermore, because of the development of acute respiratory distress syndrome (ARDS), 5% of Covid-19 affected persons may be serious cases and require help with ventilation (Al-Kuraishy et al. 2021a).

The genetic similarity of coronaviruses of Middle East (MERS-CoV) and the Covid-19 is 80 and 60%, respectively, so they are well matched with one another (Al-Kuraishy et al. 2021e). Besides, the close similarity of SARS-CoV-2 to **bat** CoV by 96% was detected at at the genomic level (Al-Kuraishy et al. 2021b). However, SARS-CoV-2 has a 20-fold more binding affinity for ACE2 than other CoVs, which gives rise to a reduction of efficient receptors (Al-Kuraishy et al. 2021e). Angiotensin II (Ang II), a vasoconstrictor, is transformed by the enzyme ACE2 to the vasodilators Ang1-7 and Ang1-9. Consequently, the SARS-CoV-2 infection causes vasoconstriction leading to the advance of oxidative stress, inflammatory diseases, and endothelial dysfunction (ED) (Al-Kuraishy et al. 2021g). These pathophysiological changes produce hypoxemia, and immune system overreaction, and lead to systemic and cardiac outcomes (Al-Kuraishy et al. 2022a).

Different drugs and agents have been repurposed in managing Covid-19 since the emergence of this pandemic (Al-Kuraishy et al. 2020b). Nevertheless, using these repurposed drugs like hydroxychloroquine and azithromycin is accompanied by adverse effects (Lane et al. 2020). Systematic reviews also confirmed the ineffectiveness of most repurposed drugs in managing Covid-19 (Kamarullah et al. 2021). Interestingly, the appearance of corona virus variants with recurrent infection, even in vaccinated persons, needs more searches to find harmless and efficient drugs or agents against this virus infection (Tay et al. 2022). The current study focusses on one of these agents, which is obeticholic acid (OCA), which was previously reported to possess anti-inflammatory effects against various intestinal and liver diseases (Chen et al. 2016). Thus, this perspective aimed to focus on the possible therapeutic efficacy of obeticholic acid (OCA) in managing Covid-19 infection.

## Pharmacology of obeticholic acid (OCA)

**Fig. 1:**
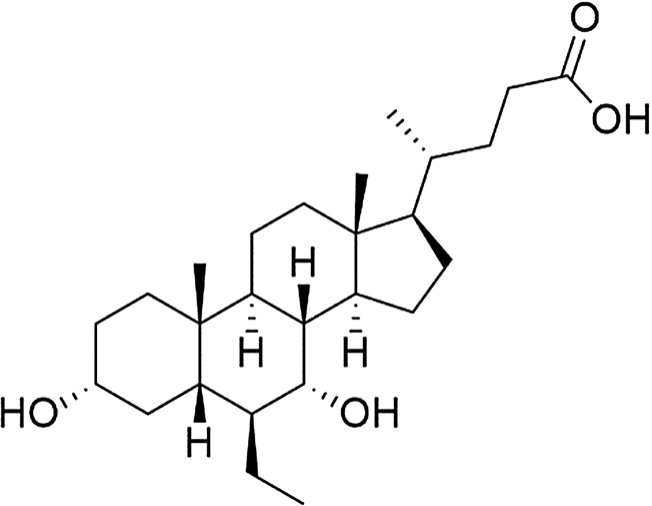
Chemical structure of obeticholic acid (OCA)

As illustrated in Fig. 1, OCA is a dihydroxy-5-β-cholanic acid, a synthetic derivative of bile acid that works as a natural legend for farnesoid X receptor (Markham and Keam 2016). OCA lessens liver exposure to the impact of bile acids (Nevens et al. 2016). In addition, it binds and activates FXRs in the intestine and liver, leading to anti-inflammatory and anti-fibrotic impacts with modulation of metabolic profiles. It also inhibits the production of bile acids and increases their transport outside the hepatocytes (Chapman and Lynch 2020). Activation of FXRs by OCA is 100 times higher than that exerted by chenodeoxycholic acid in attenuating intestinal and hepatic inflammation and/or fibrosis (Fiorucci et al. 2019). Through modulation of bile acid homeostasis, OCA effectively reduces cholestasis-induced liver inflammation/injury. Thus, OCA is prescribed in managing primary biliary cholangitis, liver cirrhosis, portal hypertension, and non-alcoholic liver inflammatory diseases (Hirschfield et al. 2015; Neuschwander-Tetri et al. 2015).

OCA is highly absorbed from the small intestine when taken orally, and its maximum plasma level is reached within 1.5 h with a biological half-life of about 24 h (Edwards et al. 2017). OCA exerted a high volume of distribution expected at 618 L. It is mostly conjugated with taurine and glycin, processed by the liver, and excreted by the bile (Valluri et al. 2021). Conjugated OCA in the intestines is reabsorbed by enterohepatic circulation, where the bacterial flora of intestine participates in deconjugation. The reabsorption or elimination is done through feces of the deconjugated form (Edwards et al. 2016).

**Fig. 2:**
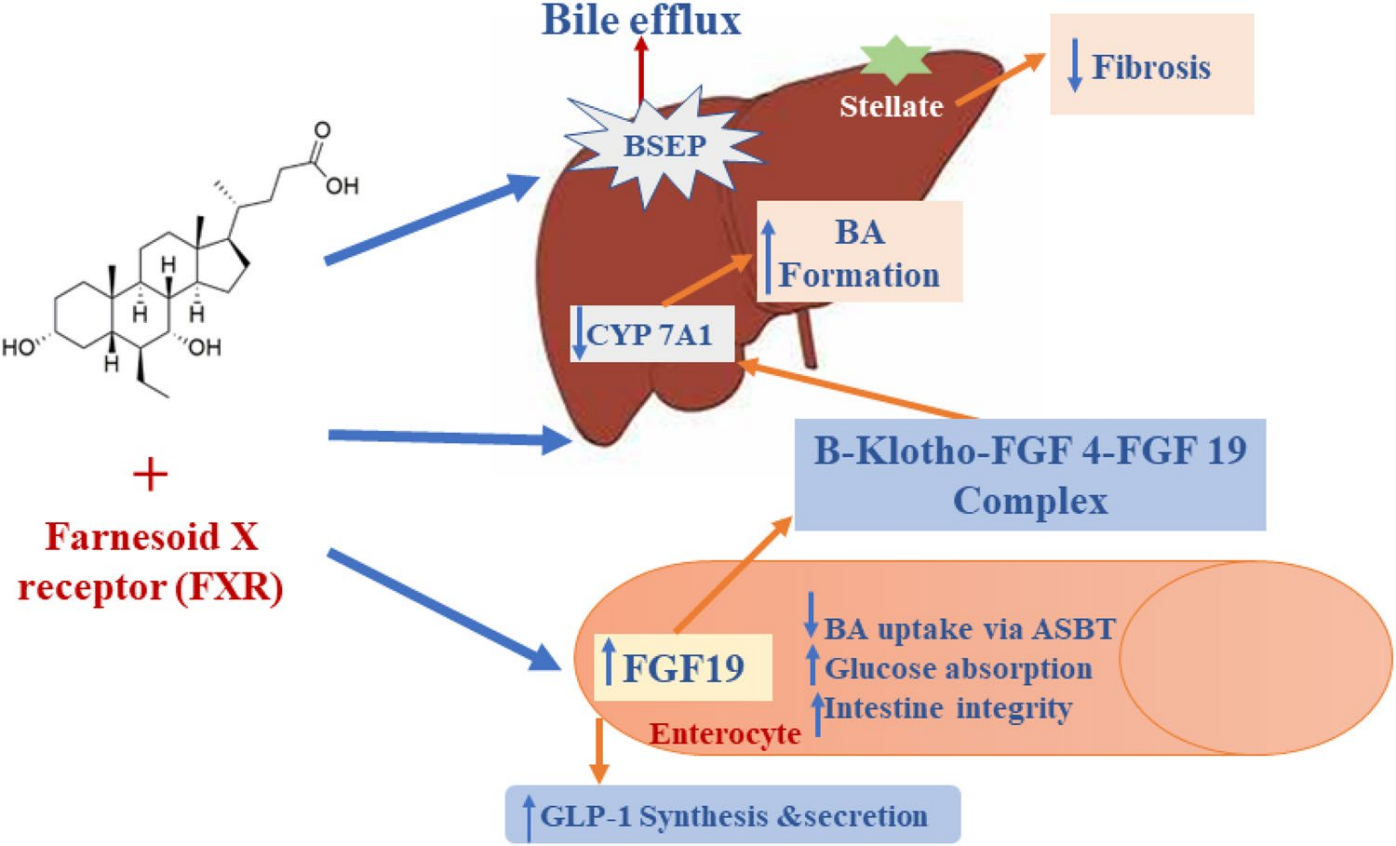
Mechanism of action of OCA in attenuating intestinal and hepatic inflammation and/or fibrosis

Moreover, OCA activitites FXR to induce the release of fibroblast growth factor 19 (FGF19) from the ileum, and downregulating the expression of hepatic CYP7A1 in the synthesis of bile acid (Edwards et al. 2017; Valluri et al. 2021). In addition, OCA enlarges the expression of bile salt exporter protein (BSEP) and multidrug resistance 3 (MDR3) and permits bile acid efflux from the hepatocytes (Valluri et al. 2021). It also exerted an oppressive effect on transforming growth factor beta (TGF-β) expression and hepatic stellate cell activation (Edwards et al. 2017; Valluri et al. 2021), where OCA‘s net mode of action is illuminated in Fig. 2.

## Anti-inflammatory effects of OCA

OCA displayed a strong anti-inflammatory impact through the decrease of inflammatory signaling pathways in lipopolysaccharide (LPS)-induced ALI in mice (Fei et al. 2019). Through stimulation of FXRs, OCA suppresses the expression of nuclear factor kappa B (NF-κB), p38 mitogen-activated protein kinase (p38MAPK), and Akt phosphorylation (Fei et al. 2019). It motivates the discharge of the anti-inflammatory cytokine, downregulating the expression of pro-inflammatory cytokines (Verbeke et al. 2016). It is worth stressing that FXR notably weakens hepatic inflammation by supressing the expression of NF-κB (Verbeke et al. 2016). An experimental study previously conducted on OCA explored that it lessened liver damage triggered by thioacetamide and disallowed the progression of portal hypertension in rats (Verbeke et al. 2016). Meanwhile, FXR knockout mice had a serious risk of evolving liver inflammation and fibrosis (Yang et al. 2007). Besides, OCA exerted a suppression effect against oxidative stress and inflammation that, in turn, weakens acute kidney damage by sepsis (AKI) in mice (Zhu et al. 2018). Acute kidney injuries caused by lipopolysaccharides in mice experiment showed that OCA administration counteracts the progression of renal inflammation and dysfunction by decreasing the levels of chemokines and pro-inflammatory cytokines associated by supressing lipid peroxidation and NADPH oxidase activity (Zhu et al. 2018).

In clinical settings, OCA efficiently lessens the inflammatory alterations noticed in diabetic patients through the modulation of lipid and glucose metabolisms (Mudaliar et al. 2013). Moreover, by stimulating nuclear hormone receptors, OCA could control insulin sensitivity and lipid metabolism in affected persons with hepatic steatosis (Mudaliar et al. 2013). A placebo-controlled trial including patient with non-alcoholic fatty liver disease (NAFLD) displayed that treatment with variable doses of OCA led to the dose-dependent effect of OCA in the downregulating of liver fibrosis and inflammation (Mudaliar et al. 2013). Therefore, OCA could be a suggested drug for treating diabetic patients with NAFLD. Additionally, a placebo-controlled trial phase 3 included 217 humans suffering from primary biliary cholangitis and disclosed that treatment with OCA 10 mg/day with ursodiol or as a monotherapy for 1 month showed a significant decrease of liver inflammation (Nevens et al. 2016). It was found that OCA modulated the inflammation and immune response in persons suffering from biliary cholangitis because it displayed anti-inflammatory and anti-fibrotic effects (Liver 2009).

**Fig. 3:**
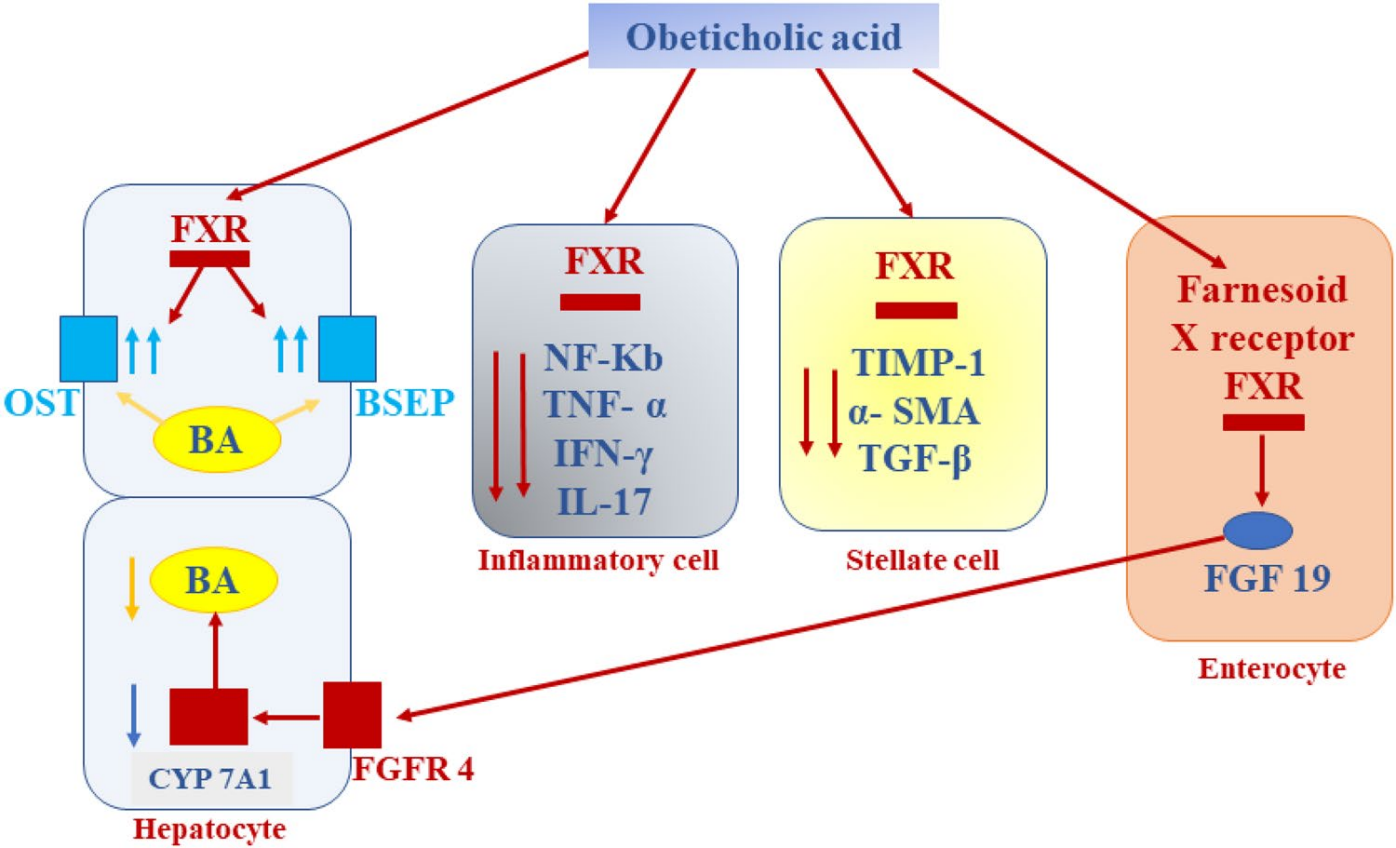
The anti-inflammatory effects of obeticholic acid (OCA), where OCA inhibits the release of cytokines that promote inflammation from immune cells. It reduces the expression of TGF-β, tissue inhibitor of metalloproteinase 1 (TIMP-1) and alpha-smooth muscle actin (α-SMA) through activation of FXR

The role of OCA against the process of inflammation is mainly arbitrated through the activation of FXRs, which are massively expressed in the intestine and liver (Fei et al. 2019). Activated FXRs are translocated to the nucleus and bind DNA hormone response elements leading to reduction of cholesterol 7 alpha-hydroxylase and stimulation of small heterodimer partner (SHP), an intracellular transcription factor of the nuclear receptor family (Yuk et al. 2016). SHP controls innate immune reaction and inflammation through supression of the production of toll-like receptor 4 (TLR4) and nod-like receptor pyrin 3 (NLRP3) inflammasome (Yuk et al. 2016). In addition, SHP stops the translocation of NF-κB p65 from the cytoplasm and suppresses the release of cytokines that promotes inflammation. (Yuk et al. 2011). Activating FXRs may block cisplatin-induced AKI via stimulation of SHP in mice (Bae et al. 2014). It has been detected that fenofibrate provokes the expression of SHP in the macrophages and hepatocytes via activation of adenosine monophosphate protein kinase (AMPK) (Chanda et al. 2009). Fenofibrate inhibits OS and inflammation by activating peroxisome proliferator activator receptor alpha (PPARα) (Enright et al. 2020). Notably, FXRs activate the expression of anti-inflammatory PPARα, and thus supress pro-inflammatory cytokines (Heitel et al. 2020). Furthermore, FXRs provoke the expression of cystic fibrosis transmembrane conductance regulator (CFTR), which controls intestinal homeostasis (Mroz et al. 2014). Harwood et al. (Harwood et al. 2021) formerly displayed that the activation of CFTR reduced lung inflammation in patients with cystic fibrosis. Meanwhile, mutation of CFTR provokes the expression and discharge of cytokines that promote inflammation (Mueller et al. 2011). Besides, OCA inhibits the discharge of pro-inflammatory cytokines from immune cells. It lessens the expression of TGF-β, tissue inhibitor of metalloproteinase 1 (TIMP-1), and alpha-smooth muscle actin (α-SMA) via activation of FXR (Khanna and Jones 2017) (Fig. 3). Thus, OCA could probably induce anti-inflammatory effects with modulation of immune response via stimulation of SHP, PPARα, and CFTR.

## Antiviral effects of OCA

The role of OCA to antagonize inflammation and the reactive oxygen species may reveal marked antiviral effects. Notably, OCA suppresses the proliferation of human immunodeficiency virus 1 (HIV-1) and its associated liver fibrotic alterations (New-Aaron et al. 2020). Therefore, OCA can invert HIV-1-induced pro-fibrotic alterations in the liver. In addition, OCA stops HIV-1 particle accumulation within the liver cells (Zhou et al. 2019). A recent study displayed that FXR agonists potentially affect the proliferation of the hepatitis B virus (HBV) (Erken et al. 2021). A double-blind placebo-controlled trial of 73 cases with HBV infection treated with FXR agonists for 35 days demonstrated that these agents efficiently lessened the hepatitis B surface antigen (HBsAg) level relative to the placebo impact (Erken et al. 2021). FXR agonists interact with HBV viral proteins preventing their transcription and triggering off the reduction of HBV viral protein (Erken et al. 2021). These findings may explain the role of FXR agonists against viral infections, including OCA, to antagonize HBV infection.

Likewise, Kim et al. (Kim and Chang 2011) realized that FXR agonists supress the proliferation of rotavirus through modulation of intracellular lipid homeostasis. Notably, FXR agonists inhibit the entry of HCV by modulating scavenger receptor class B type 1(SR1B) expression with concurrent disturbance of the HCV life cycle (Wu et al. 2019b). OCA as a FXR agonist effectively inhibited the expression of SR1B in mice with hypercholesterolemia (Dong et al. 2019). Moreover, FXR agonists may indirectly interfere with viral replication by increasing SHP expression (Bae et al. 2014). SHP reduces the expression and interaction of HCV NS5A protein with the hepatocytes (Conti et al. 2016). However, an experimental study showed that SHP is implicated in the occurrence of abnormal lipid and glucose homeostasis during HCV infection (Chen et al. 2019). Therefore, induced SHP by FXR agonists may have beneficial and detrimental effects depending on the stages and types of viral infections. Besides, epigallocatechin inhibits the proliferation of HBV through stimulation of FXR (Xu et al. 2016).

On the other side, FXRs agonists provoke the generation of anti-inflammatory PPARα (Heitel et al. 2020), which displays antiviral effects against HCV and HBV (Jiang et al. 2019; Negro 2009). Read et al. found that PPARα agonists improve interferon response during HCV infection (Read et al. 2015). Similarly, OCA can regulate the immune response during viral infection with an enhancement of viral clearance via the enhancement of SHP expression (Kim et al. 2019; Yuk et al. 2016). Through the enhancement of AMPK, SHP could reduce viral load (Chanda et al. 2009) and prevent the development of different types of viral infections, including flavivirus (Jiménez de Oya et al. 2018) and Zika virus (Singh et al. 2020) infections. Consequently, these verdicts suggest that FXR agonists comprising OCA may have potential antiviral properties.

## OCA and SARS-CoV-2 infection

The direct action with viral proteins and binding with ACE2 or indirectly via modulation of immune response and inflammatory reactions could be the mode of action of OCA to manage the course of Covid-19 (Carino et al. 2020). Primary and secondary bile acids and their derivatives and semisynthetic bile acid-like OCA block the interaction between SARS-CoV-2 and ACE2 in vitro; thereby supressing the entrance of SARS-CoV-2 and the progression of Covid-19 (Carino et al. 2020). An additional ex vivo study by Choi et al. discovered that OCA in a concentration of 0.98 nM supressed the reaction between SARS-CoV-2 and ACE2/TMPRSS2 axis (Choi et al. 2020). Additionally, in silico study disclosed that OCA reduced the reaction of SARS-CoV-2 with ACE2 and further consolidated the possible antiviral impact of OCA (Sibilio et al. 2021). Rigamonti et al. disclosed five cases of autoimmune liver diseases with Covid-19 and confirmed that OCA management with other supportive treatments ameliorates patients' clinical outcomes (Rigamonti et al. 2020). However, long-term treatment of OCA in ill persons with autoimmune liver diseases may weaken adverse events of SARS-CoV-2 (Al-Kuraishy et al. 2021d). The progression of autoimmune liver diseases could be provoked via the SARS-CoV-2 vaccine (Gómez-Domínguez et al. 2022). OCA utilization in liver fibrosis induced by Covid-19 was not supported (Wu et al., 2020). Nevertheless, the SARS-CoV-2 infection risk and its accompanied inflammatory reactions could be weakened by OCA treatment in patients suffering from autoimmune liver diseases (Hamid et al. 2021; Sibilio et al. 2021).

## The possible mechanisms of OCA in Covid-19

The serious manifestations of Covid-19 could be controlled by the anti-inflammatory, antioxidant, and antiviral effects of OCA. These impacts are chiefly triggered through the activation of FXRs, which are greatly expressed in different cells (Fei et al. 2019). OCA suppresses the expression of NF-κB, p38MAPK, and Akt phosphorylation through provoking FXRs (Fei et al. 2019). Additionally, it facilitates the discharge of anti-inflammatory cytokines accompanied by inhibition of pro-inflammatory cytokines expression (Verbeke et al. 2016). It is worth stressing on severe SARS-CoV-2 infection, some small proteins with significant role for the growth and activity of immune cells like IL-4 and IL-10 are decreased while pro-inflammatory cytokines like IL-1β, IL-6, and 1L-17 are upregulated with concomitant progression of hypercytokinemia (Al-Kuraishy and Al-Gareeb 2021; Al-Kuraishy et al. 2022a, 2021f). Moreover, NF-κB, p38MAPK, and high mobility group box-1 (HMGB1) are strongly stimulated in serious SARS-CoV-2 and accompanied with the progression of ALI/ARDS and thrombotic events (Al-Kuraishy et al. 2022b, 2022c).

In addition, FXR agonists inhibit the triggering of nod-like receptor pyrin 3 (NLRP3) inflammasome, which is included in the overstated immune response and propagation of hypercytokinemia in severe SARS-CoV-2 infection (Batiha et al. 2021; Lu et al. 2022). In this sense, OCA via provoking of FXRs may lessen the risk of hyperinflammation and production of hypercytokinemia, in patients with severe Covid-19. It was disclosed that SARS-CoV-2 infection is also associated with the emergence of OS, because of the production of reactive oxygen species (ROS) and the decreased endogenous antioxidant capacity (Mostafa-Hedeab et al. 2022). Indeed, NADPH oxidase is directly provoked in SARS-CoV-2 infection, resulting in OS development (DiNicolantonio and McCarty 2020). Basically, OS in Covid-19 triggers the discharge of cytokines that trigger inflammation with subsequent production of hypercytokinemia (Derouiche 2020). In turn, hypercytokinemia and hyperinflammation trigger the propagation of OS (Meftahi et al. 2021), whereas both OS and hyperinflammation in SARS-CoV-2 infection are greatly implicated and correlated with the advancement of thrombotic events in Covid-19 patients (Fodor et al. 2021). Notably, OCA suppresses the discharge of chemokines and pro-inflammatory cytokines with the inhibition of lipid peroxidation and NADPH oxidase (Zhu et al. 2018).

Furthermore, OCA has a potent antagonistic effect against inflammation by inhibiting the discharge of cytokines that promote inflammation in persons suffering from primary biliary cholangitis (Chapman and Lynch 2020). Likewise, OCA weakens the progression of OS by suppressing NADPH oxidase, ROS generation, lipid peroxidation, and stimulating antioxidant enzymes in LPS-induced ALI in mice (Gai et al. 2020; Zhu et al. 2018). Wu et al. found that OCA protects against the development of diabetic cardiomyopathy through the stimulation of antioxidant nuclear factor erythroid-derived 2 (Nrf2) in mice (Wu et al. 2019a). Thus, OCA could serve as an effective agent in blocking SARS-CoV-2 infection-mediated OS and hyperinflammation and its associated complications.

## FXR and Covid-19

In general, FXR participates with a likely role in different forms of viral infections, including SARS-CoV-2. It was discovered that FXR upregulated the expression of ACE2 in the affected tissues, including gastrointestinal and respiratory systems, and probably permitted SARS-CoV-2 cell entry (Brevini et al. 2021a). Ursodeoxycholic acid (UDCA), which modulates FXR expression, lessens circulating ACE2 levels in vivo, thereby it decreases the severity of hospitalized Covid-19 patients (Brevini et al. 2021a). However, UDCA reduces airway inflammation through the modulation of FXR expression and the development of eosinophilic inflammation (Thuy et al. 2022). Brevini and colleagues revealed that FXR antagonist O07 could effectively manage Covid-19 (Brevini et al. 2021b). Ex vivo and in vitro data demonstrated that FXR antagonist O07 could be beneficial chemoprophylaxis against developing SARS-CoV-2 infection via inhibition expression of ACE2 (Brevini et al. 2021b).

Meanwhile, guggulsterone, a FXR antagonist, displayed immunomodulatory effects and can lessen the risk of hypercytokinemia, in obese ill persons suffering from Covid-19 (Preethi et al. 2021). Despite in vitro and in vivo findings approving the beneficial effects of FXR antagonists in ameliorating the harshness of SARS-CoV-2 infection, this effect might be regulated by drug-specific effects rather than blocking the FXR effect. Meanwhile, FXRs have anti-inflammatory and antioxidant effects, whereas FXR-induced expression of ACE2 is beneficial rather than harmful (Verbeke et al. 2016; Yang et al. 2007).

Interestingly, angiotensin receptor blockers (ARBs) and angiotensin-converting enzyme inhibitors (ACEIs), which increase ACE2 expression, were initially involved in the pathogenesis of SARS-CoV-2 infection, and seem nowadays to be defensive against Covid-19 severity (Thomas et al. 2022). Similarly, ibuprofen which upregulates ACE2 expression displayed a defensive effect against Covid-19 infection (Poutoglidou et al. 2021). Furthermore, soluble recombinant ACE2 could be efficient to antagonize the severity of SARS-CoV-2 infection by downregulating the pro-inflammatory angiotensin II (AngII) with concomitant elevation of anti-inflammatory angiotensin (1–7) (Ang1-7) (Zhang et al. 2021). Therefore, linking ACE2 expression with SARS-CoV-2 infection should be reconsidered, and this pathway might not regulate the efficacy of FXR antagonists in Covid-19.

On the other hand, FXR agonists like cafestol, chenodeoxycholic acid, fexaramine, ivermectin, and tropifexor, in addition to OCA, may play a critical role in SARS-CoV-2 infection (Carotti et al. 2014). It has been demonstrated that increasing bile acid production under high body temperature promotes the generation of chenodeoxycholic acid from gut microbiota. In addition, chenodeoxycholic acid limits SARS-CoV-2 proliferation and associated tissue injury in mice through activation of FXR (Babalghith et al. 2022). Notably, through modulation of bile acid metabolism, gut microbiota provokes the stimulation of anti-inflammatory FXR (Hollman et al. 2012; Zhang et al. 2013) with following supression of the proliferation of the virus of Covid-19 (Spagnolello et al. 2021). Furthermore, ivermectin displayed strong antiviral and anti-inflammatory effects through the stimulation of FXR (Low et al. 2022). Interestingly, FXRs are greatly deregulated in Covid-19 patients due to OS and immune system overreaction (Alaiya et al. 2021). Therefore, the supressing effects against immune system overreaction and ROS of FXR agonists may lessen the harmful effects of Covid-19 and accompanied complications.

## FXR and signaling pathways in Covid-19

The anti-inflammatory effect of FXR agonists is regulated via provoking of SHP, PPARα, and CFTR, decreasing the expression of cytokines that provokes immune system reaction (Heitel et al. 2020; Mueller et al. 2011; Yuk et al. 2016). The innate immune reaction and inflammation could be regulated by SHP through supressing the expression of TLR4, NLRP3 inflammasome, and NF-κB (Yuk et al. 2016, 2011). Notably, TLR4, NLRP3 inflammasome, and NF-κB are highly stimulated in SARS-CoV-2 infection resulting in hyper-inflammation and hypercytokinemia, (Batiha et al. 2021; Lu et al. 2022). SHP affords a negative regulatory effect on various signaling pathways. For example, it decreases virus-mediated interferon signaling and innate immune response through interaction with CREB-binding protein (CBP) (Kim et al. 2019). The immunosuppressive effect of SHP may enhance the viral infection, but at the same time it weakens the augmentation of immune response (Kim et al. 2019) as well as the progression of hypercytokinemia, a hallmark of Covid-19 severity (Jiang et al. 2022). Supression of CBP by glycogen synthase kinase 3 (Gsk-3) promotes the progression of systemic inflammation and OS in severe SARS-CoV-2 infection (Rana et al. 2021). In this state, activating CBP or inhibiting Gsk-3 could be beneficial in preventing Covid-19 severity. Thus, triggering of SHP pathway by FXR agonists like OCA may reduce immunoinflammatory disorders in Covid-19 patients.

Furthermore, FXR agonists can provoke the expression of PPARα, which possesses potent immunomodulatory effects in SARS-CoV-2 infection (Fantacuzzi et al. 2022). PPARα agonists can lessen pulmonary inflammation, lipotoxicity, and metabolic derangement induced by SARS-CoV-2 infection (Fantacuzzi et al. 2022). Besides, in vitro study displayed that fenofibrate inhibits SARS-CoV-2-mediated cytopathic in Vero E6 cell lines at a concentration of 20 µM (Rodon et al. 2021). Yasmin et al. (2022) suggested that fenofibrate attenuates the interaction between SARS-CoV-2 and ACE2. PPARα agonists supress the activation of inflammatory signaling pathways and the discharge of pro-inflammatory cytokines (Fantacuzzi et al. 2022). Therefore, direct PPARα agonists and indirect stimulation of these receptors by FXR agonists may represent promising treatments when included in the Covid-19 therapeutic protocols (Fantacuzzi et al. 2022).

Moreover, FXR agonists, upregulating the expression of cystic fibrosis transmembrane conductance regulator (CFTR), may modulate the pathogenic course and immunological response during SARS-CoV-2 infection. It has been disclosed that CFTR is greatly downregulated in SARS-CoV-2 infection with the development of acquired cystic fibrosis in Covid-19 (Lidington and Bolz 2020). CFTR is expressed in many critical organs, including the intestines, lungs, brains, pancreas, kidneys, blood vessels, and immune cells (Lara-Reyna et al. 2020). High pro-inflammatory cytokines, mainly TNF-α, are chiefly imposed in the downregulation of CFTR in the brain and lung (Yagi et al. 2015). Thus, exaggerated TNF-α levels in Covid-19 could be the causative factor behind the deregulation of CFTR. In this state, deregulated CFTR could engage in respiratory and other systemic complications in Covid-19 patients (Lidington and Bolz 2020).

Notably, CFTR has a critical role in regulating immune response, as different immune cells, like macrophages, monocytes, and neutrophils, express these receptors (Zhang et al. 2018). Loss or dysfunction of CFTR promotes macrophage activation and release of pro-inflammatory cytokines (Zhang et al. 2018). Activation of CFTR could be beneficial in damping exaggerated immune responses by inhibiting the release of pro-inflammatory cytokines (Zhang et al. 2018). Interestingly, CFTR agonists like Trikafta are expensive and cannot be used widely (Lidington and Bolz 2020). Therefore, indirect activation of CFTR by FXR agonists like OCA could be beneficial. FXR agonists via increasing the expression of CFTR may reduce immunoinflammatory and pulmonary disorders in Covid-19 patients. In addition, FXR agonists like OCA may reveal direct effects in the modulation of immune response in SARS-CoV-2 infection or indirect effect through activation of SHP, PPARα, and CFTR which prohibit the expression of pro-inflammatory cytokines. Herein, experimental, preclinical, and clinical studies are needed in this regard.

In 2016, obeticholic acid (Ocaliva™) was awarded approval to utilize OCA in patients with primary biliary cirrhosis (PBC) who are UDCA intolerant or their health does not get better by treatment with UDCA after a year. In the dose-dependent clinical studies of two phases, using dosages of OCA up to 50 mg per day, pruritus was the frequently observed adverse effect of OCA. Clinical studies of 217 UDCA nonresponders or UDCA intolerant participants in the pivotal phase 3 trial were randomly subjected to 1 of 3 treatments: placebo, OCA 5 mg/day with dose titration to 10 mg if necessary, or OCA 10 mg/day. Intercept Pharmaceuticals nowadays develops Ocaliva™, a FXR agonist, to manage different liver diseases (Chapman and Lynch 2020). Ocaliva™ received accelerated approval in the USA for managing primary biliary cholangitis which is combined with ursodeoxycholic acid in adults who have a weak response to ursodeoxycholic acid or as monotherapy in adults who cannot tolerate ursodeoxycholic acid.

To this point, FXR agonists supress the entrance of HCV by modulating the scavenger receptor class B type 1(SR1B) expression by disturbing the HCV life cycle (Wu et al. 2019b). FXR agonist OCA suppresses the expression of SR1B in mice with hypercholesterolemia (Dong et al. 2019). SR1B facilitates the entry of SARS-CoV-2 through ACE2. Thus, a monoclonal antibody against SR1B reduces the severity of SARS-CoV-2 (Wei et al. 2020). In addition, SR1B expressed in the immune cells provokes the discharge of pro-inflammatory cytokine and the development of autoimmune diseases (Wei et al. 2020). Therefore, inhibition of SR1B may attenuate SARS-CoV-2 entry and release of pro-inflammatory cytokine.

## Conclusion

In serious SARS-CoV-2 infection, some cytokines that provoke inflammation like IL-4 and IL-10 are lessened, and at the same time, pro-inflammatory cytokines such as IL-1β, IL-6, and 1L-17 are upregulated with concurrent development of hypercytokinemia. Furthermore, SARS-CoV-2 infection is related to the development of OS due to ROS generation and reduction of endogenous antioxidant agents. Indeed, NADPH oxidase is directly stimulated in SARS-CoV-2 infection, causing OS progression. OS in SARS-CoV-2 infection triggers the discharge of pro-inflammatory cytokine and the development of hypercytokinemia. In turn, hypercytokinemia and hyperinflammation provoke the proliferation of OS. Both OS and hyperinflammation in SARS-CoV-2 infection are interrelated in advancing thrombotic events in ill persons suffering from Covid-19. Therefore, supressing the augmented immune system reaction and reactive oxygen species by agents like OCA may weaken OS and inflammatory disorders in Covid-19 patients. OCA is a FXR agonist that controls immunoinflammatory alterations and is induced by SARS-CoV-2 infection. FXR agonists regulate the expression of ACE2 and inflammatory signaling pathways in Covid-19, which weakens the severity of SARS-CoV-2 infection and related complications. Taken together, FXR agonists like OCA may reveal both direct and indirect effects in the modulation of immune response in SARS-CoV-2 infection. Thus, experimental, preclinical, and clinical studies are necessary and highly recommended.

## Data Availability

The authors confirm that the data supporting this study are available within the article.
